# Supplementary material for: Prioritisation of ocean biodiversity data collection to deliver a sustainable ocean
Source: Commun Earth Environ. 2025 Jun 18;6(1):473. doi: 10.1038/s43247-025-02442-7 (PMC12176621; doi:10.1038/s43247-025-02442-7)
Supplement: Supplementary file 1 — Supplementary information [file 43247_2025_2442_MOESM1_ESM.pdf]

SUPPLEMENTARY INFORMATION

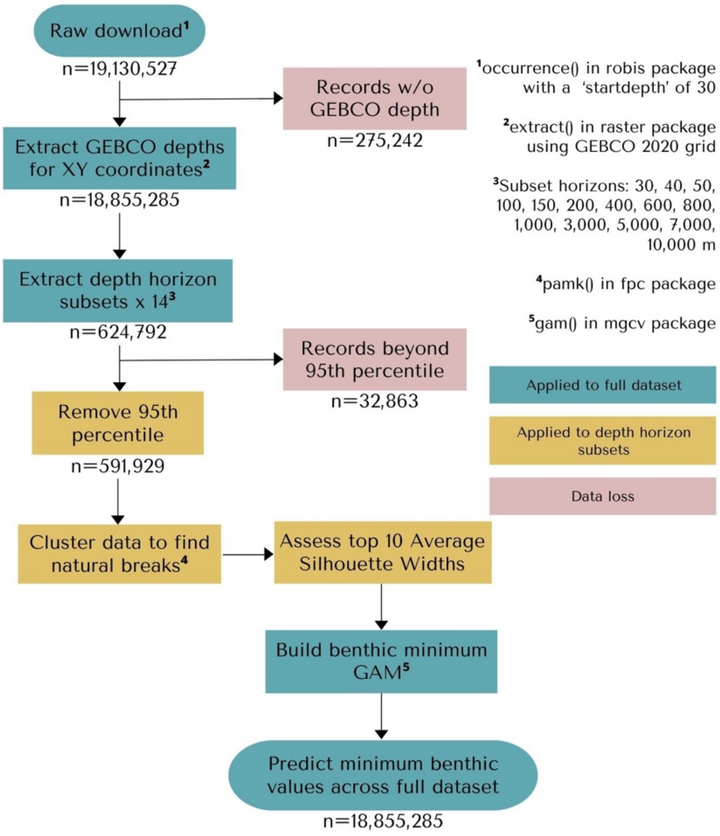

Figure S1: Data pipeline developed to extract a clean, benthic dataset from the Ocean Biodiversity Information Service.

Table S1: Depth horizons at which data were subset to identify the benthic data point cloud.

Depth horizon subset	No. of records	No. of records after the removal of 95th percentile
30	89,922	85,399
40	238,845	226,740
50	91,277	86,031

100	59,827	56,363
150	40,503	38,464
200	13,934	13,181
400	13,095	12,423
600	4,352	4,114
800	3,176	3,011
990-1,010	48,834	46,392
2,990-3,010	13,535	12,848
4,990-5,010	7,293	6,783
6,990-7,010	49	39
9,990-10,010	150	141

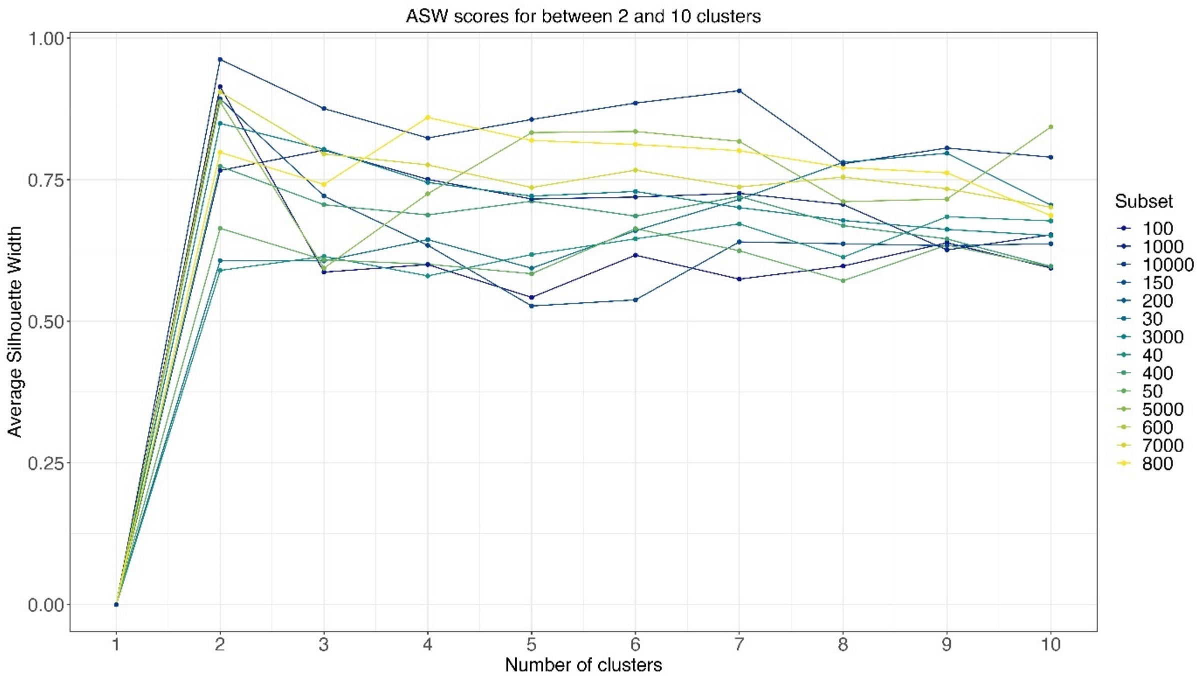

Figure S2: Average Silhouette Widths (ASWs) for between 2 and 10 clusters per subset.

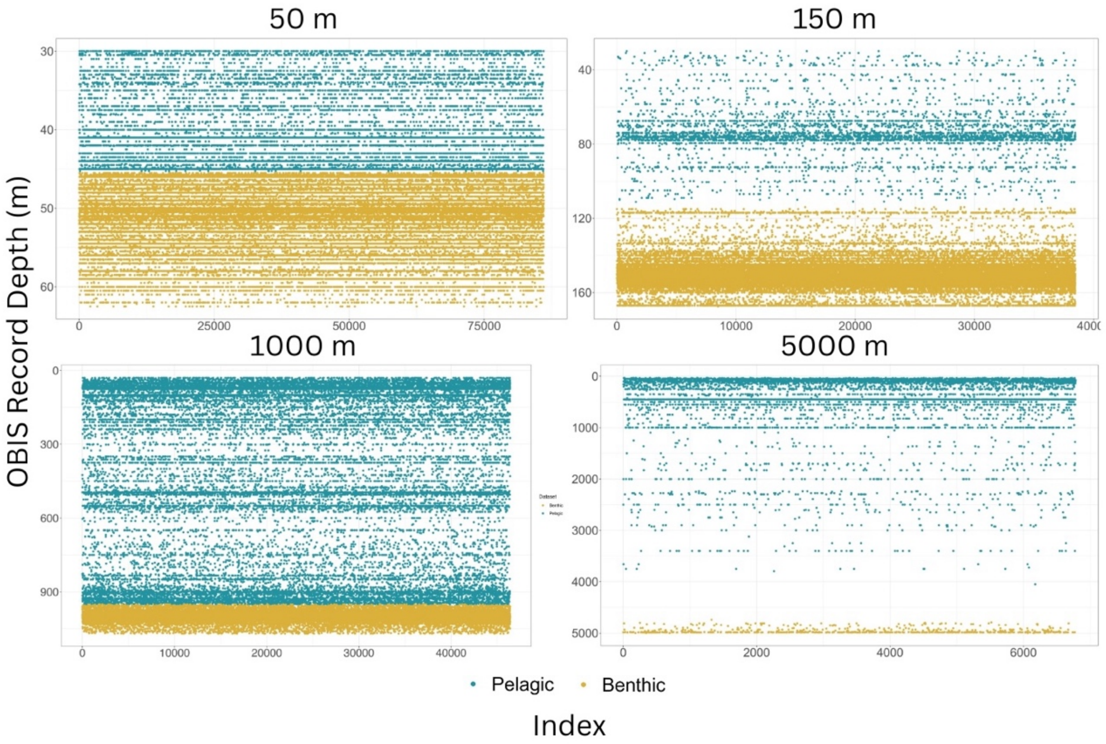

Figure S3: Four data subsets where benthic and pelagic points are indicated.

Table S2: Selected cluster details for each of the 14 depth horizon subsets.

Depth horizon subset	Benthic minimum depth (m)	Benthic maximum depth (m)	No. of clusters applied on subset	Average Silhouette Width	Ranked solution (1 to 10)
30	30	33.5	2	0.61	7
40	38.6	42.1	3	0.61	6
50	45.5	62.5	2	0.66	1
100	74	109.5	2	0.91	1
150	114	167.5	2	0.89	1
200	174	229	5	0.71	3
400	300	444	3	0.91	4
600	554	659	5	0.82	2
800	777	825	4	0.72	1
990-1,010	951	1,070	4	0.75	3
2,990-3,010	2,540.99	3,057.26	6	0.73	4
4,990-5,010	4,740	5,000	6	0.84	3
6,990-7,010	6,102.5	7,000	4	0.78	3
9,990-10,010	9,676.5	10,898	2	0.96	1

Table S3: The generalised additive model run on the 14 benthic minima values to model the benthic data cloud.

	Benthic minimum GAM
Formula	obis_benthic_min ~ s(gebco_depth_value)
Estimated intercept value	1,865.578
Estimated degrees of freedom	8.266
F-statistic	98,627
Generalized Cross Validation (GCV)	386.6

Table S4: Modelled upper limit of the benthic data cloud for the 14 predetermined depth subset values and associated 95% confidence intervals.

Depth horizon subset	Modelled upper limit of the benthic data cloud (m)	95% upper confidence limit (m)	95% lower confidence limit (m)
30	28.23	14.72	41.75
40	36.24	23.99	48.48
50	44.23	33.07	55.39
100	83.64	73.64	93.64
150	121.13	108.18	134.08
200	156.40	141.62	171.17
400	311.33	291.34	331.31
600	549.21	529.14	569.28

800	775.53	755.10	795.97
1,000	952.11	930.16	974.06
3,000	2541.14	2518.74	2563.55
5,000	4739.82	4717.42	4762.23
7,000	6102.61	6080.21	6125.02
10,000	9691.83	9669.32	9714.35

Table S5: Number of OBIS records remaining at key stages of the data pipeline.

Pipeline step	No. of records	% of raw data
All OBIS records from 30 m+	19,130,527	100
Records without GEBCO depth	275,242	1.4
Records with GEBCO depth	18,855,285	98.6
Predicted benthic records	12,656,103	66.2
Predicted pelagic records	6,198,683	32.4

Supplementary Notes 1

Fitting a GAM to the 14 benthic minimum values derived from clustering resulted in a highly effective model that, using GEBCO depth as the predictor ($p < 0.001$), explained 100% of deviance (Supplementary Table 4). The smooth term exhibited a notable relationship with the predicted response variable suggesting a non-linear association between GEBCO depth and

benthic minimum observations. Given we know that error in depth records increases with increasing depth, this is to be expected.
